# Artificial Intelligence for Identification of Images with Active Bleeding in Mesenteric and Celiac Arteries Angiography

**DOI:** 10.1007/s00270-024-03689-x

**Published:** 2024-03-26

**Authors:** Yiftach Barash, Adva Livne, Eyal Klang, Vera Sorin, Israel Cohen, Boris Khaitovich, Daniel Raskin

**Affiliations:** 1 Department of Diagnostic Imaging, Chaim Sheba Medical Center, Emek Haela St. 1, 52621 Ramat Gan, Israel; 2 The Faculty of Medicine, Tel-Aviv University, Tel Aviv, Israel; 3 DeepVision Lab, Chaim Sheba Medical Center, Emek Haela St. 1, 52621 Ramat Gan, Israel; 4 Sami Sagol AI Hub, ARC, Chaim Sheba Medical Center, Emek Haela St. 1, 52621 Ramat Gan, Israel

**Keywords:** Artificial intelligence, Interventional radiology, Gastrointestinal bleeding, Convolutional neural networks

## Abstract

**Purpose:**

The purpose of this study is to evaluate the efficacy of an artificial intelligence (AI) model designed to identify active bleeding in digital subtraction angiography images for upper gastrointestinal bleeding.

**Methods:**

Angiographic images were retrospectively collected from mesenteric and celiac artery embolization procedures performed between 2018 and 2022. This dataset included images showing both active bleeding and non-bleeding phases from the same patients. The images were labeled as normal versus images that contain active bleeding. A convolutional neural network was trained and validated to automatically classify the images. Algorithm performance was tested in terms of area under the curve, accuracy, sensitivity, specificity, F1 score, positive and negative predictive value.

**Results:**

The dataset included 587 pre-labeled images from 142 patients. Of these, 302 were labeled as normal angiogram and 285 as containing active bleeding. The model’s performance on the validation cohort was area under the curve 85.0 ± 10.9% (standard deviation) and average classification accuracy 77.43 ± 4.9%. For Youden’s index cutoff, sensitivity and specificity were 85.4 ± 9.4% and 81.2 ± 8.6%, respectively.

**Conclusion:**

In this study, we explored the application of AI in mesenteric and celiac artery angiography for detecting active bleeding. The results of this study show the potential of an AI-based algorithm to accurately classify images with active bleeding. Further studies using a larger dataset are needed to improve accuracy and allow segmentation of the bleeding.

**Graphical abstract:**

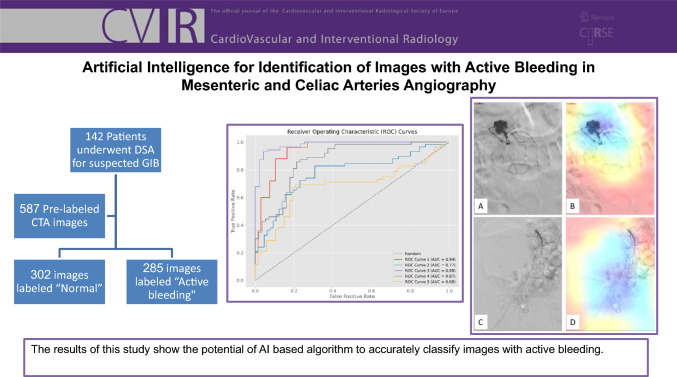

**Supplementary Information:**

The online version contains supplementary material available at 10.1007/s00270-024-03689-x.

## Introduction

Acute nonvariceal upper gastrointestinal bleeding (UGIB) represents a critical emergency, requiring immediate and accurate diagnosis and treatment. Early endoscopic evaluation, following patient stabilization, is necessary for initial UGIB management. However, for patients with ongoing hemodynamic instability, imaging modalities such as CT angiography (CTA) and catheter angiography become crucial [1]. CTA plays a vital role in localizing the bleeding source, especially when endoscopic methods are inconclusive or contraindicated. The integration of artificial intelligence (AI) in CTA has further enhanced its utility in accurately identifying bleeding sources [2–4].

In cases where endoscopy identifies the bleeding source but fails to control the bleeding, catheter angiography is recommended [5]. When endoscopy reveals bleeding without a clear source or when endoscopy is negative, catheter angiography or CTA can be used in guiding subsequent management decisions [6–8].

With transcatheter angiography, extravasation of contrast material into the bowel is a key indicator of GIB. If no signs of extravasation are observed, superselective angiography is recommended. Based on endoscopic or CTA findings that provide information about the likely location of the bleeding source, superselective catheterization of the gastroduodenal artery or left gastric artery may be performed [9]. Transcatheter arterial embolization has demonstrated significant efficacy in controlling bleeding in a substantial number of patients with GIB [10, 11], However, the detection rate of extravasation via angiography varies significantly, reported as ranging from 24% [12] to 78% [13]. This variation is influenced not only by the technical aspects of the procedure but also by the nature of GIB itself, which may spontaneously resolve and thus affect detection rates [14]. Acknowledging this complexity provides a more accurate understanding of the challenges in diagnosing GIB and the specific role of CTA in this context.

To address this challenge and provide valuable support to interventional radiologists during the procedure, we have developed an artificial intelligence (AI) model capable of identifying angiographic images with active bleeding. The aim of this study is to evaluate the diagnostic performance of this AI model in detecting extravasation on digital subtraction angiography (DSA) images in patients with UGIB.

## Methods

### Patients and DSA Imaging

This retrospective study was approved by the local institutional review board (IRB) with a waiver of informed consent. Included in the study were patients who underwent angiographic procedures in our tertiary medical center from January 2018 to December 2022. Angiography was done to identify and treat acute UGIB. DSA was performed as a standard procedure to ascertain the status of the arterial system, with particular attention given to the branches of the celiac and SMA arteries for active bleeding.

The angiographic evaluation protocol for acute gastrointestinal (GI) hemorrhage is tailored to individual patient needs while adhering to established general outlines. Access is achieved using a 5fr sheath, followed by catheterization with a cobra catheter over a 0.35 glide wire. For cases where extravasation is noted on endoscopy within the celiac branches (left gastric and common hepatic arteries), these areas are specifically targeted for selective angiography. If bleeding is identified on preprocedural CT scans, the corresponding visceral artery is catheterized. Microcatheters are employed for more selective procedures, particularly when extravasation is observed during angiography. All contrast injections are manual, and bowel stasis medications are not used in our protocol. Fluoroscopy are conducted initially on anterior–posterior (AP) view, and subsequently appropriate angulated views are used to better present the vasculature and the bleeding.

The angiographic approach begins with the selective catheterization of the artery most likely to be the source of bleeding, based on available clinical, endoscopic, and imaging data. In suspected upper GI hemorrhage, this typically involves evaluating the celiac artery first, followed by the superior mesenteric artery (SMA) as needed. The SMA and the inferior mesenteric artery (IMA) are given attention in lower GI tract evaluations, especially considering mesenteric circulatory variations. Aortography is not conducted when a preprocedural or previous imaging is present and used for procedural planning. If microcatheters are used, completion angiography is conducted from the catheter. Regardless of the origin of the extravasation, mesenteric arteries are assessed post-procedurally to make sure no other sources of hemorrhage exist.

For each patient included in the study, two radiology residents selected images displaying active bleeding and images showing a normal arterial tree. This process specifically excluded images with significant motion artifacts, ensuring high-quality data for our AI algorithm analysis.

### Image and Data Management

To compile the dataset for our study, DSA images were manually downloaded from the Picture Archiving and Communication System (PACS) server at our medical center. Once compiled, this dataset was then uploaded to our local AI server for further processing and analysis by our AI algorithm.

Prior to computational processing, a senior interventional radiologist (DR) and a senior abdominal radiologist (EK) labeled the obtained DSA images. The images were classified as normal arterial structures or images presenting arterial extravasation. Discordance was resolved by consensus. The dataset was partitioned into training and validation subsets with a ratio of 0.8 to 0.2, using fivefold cross-validation. Patients were randomly chosen for the training/validation datasets. In the study, stringent measures were implemented to prevent leakage bias, a crucial step in maintaining the integrity of the AI model evaluation. Strict patient-level differentiation was ensured between the training and validation datasets. The images included in the validation set were from different individuals than those in the training set. This approach guaranteed that the model was tested on completely unfamiliar data, thereby providing a true assessment of its performance and generalizability.

### Software and Hardware Specifications

The algorithms were developed using Python [version 3.9], leveraging the open-source TensorFlow library [version 2.10]. The scikit-learn library [version 1.0.1] facilitated the statistical analysis. Processing was executed on an AMD Ryzen Threadripper PRO 3945 WX CPU @ 4.00 GHz, RAM: 64.0 GB machine, complemented by an NVIDIA® RTX A5000 Graphics Card.

### Neural Network Model

We implemented the Efficient-Net B5 as our training model, a specialized image classification algorithm [15] recognized for being a state-of-the-art classification network. We employed transfer learning from the ImageNet repository to pre-train the model. After that, the weights of the final layer were adjusted using DSA images. The DSA images were preprocessed by reducing their size to a 456 × 456 × 3 matrix. The training of the model utilized these settings by default: 5 epochs, a batch size of 2, and optimization using ADAM with a 10^–5^ learning rate. The loss function was based on cross-entropy. The model differentiated between normal images and those depicting arterial extravasation, classifying them into binary categories.

### Class Activation Maps

Class activation maps (CAMs) were used to showcase the region in each image most contributing to the model’s classification decision [16]. For CAM generation, we employed gradient-weighted class activation mapping (Grad-CAM) [17]. This technique applies the gradients entering the final convolutional layer to construct a localization map (heatmap) that represents the maximum gradient locations. GradCAM and attention maps were produced with Jacob Gil's “pytorch-grad-cam” project [18] using the last layer of the customized Efficient-Net B5model. The GradCAM and attention map were referred to as “visualization” and “grayscale_cam”, respectively.

### Metrics

In evaluating the performance of our model, we followed a structured and comprehensive approach detailed as follows:

1. Operational Threshold Optimization: the initial step involved fine-tuning the operational threshold using the area under the receiver operating curve (AUC) as the primary optimization metric. This process was crucial for determining the most effective balance between true-positive and false-positive rates.
2. Five-fold Cross-Validation for Standard Deviation: we employed a fivefold cross-validation method to calculate the standard deviation for precision and recall. This approach provided a more robust and reliable estimation of the model’s performance across different subsets of the data.
3. Calculation of Additional Performance Metrics: Following the fivefold cross-validation, we calculated other key performance metrics, including accuracy, F1 score, precision/positive predictive value (PPV), negative predictive value (NPV), recall/sensitivity, and specificity.
4. Youden’s index computation: to encapsulate the model's discriminative power, Youden’s index was computed. This index reflects the effectiveness of our AI model in differentiating between images with and without extravasation.
5. Evaluation at fixed Sensitivities: the final step involved evaluating the model’s performance at fixed recall/sensitivities of 90%, 95%, and 99%. This evaluation was crucial to understand the model’s behavior under various clinical sensitivity requirements.

In order to quantitatively assess the inter-rater reliability of image labeling between the senior radiologists, we employed Cohen’s kappa statistic. This measure was calculated based on the initial independent labeling by the residents and the subsequent consensus labeling.

## Results

From January 2018 to December 2022, a total of 142 patients underwent angiographic procedures for diagnosing and treating UGIB. From these procedures, we collected a total of 587 DSA images. Discordance between the two senior radiologists that labeled the images was observed in 55 images, which was resolved by consensus. Of these, 302 images were classified as normal, and 285 were flagged as pathological images exhibiting arterial extravasation. The calculated Cohen’s Kappa score for inter-reader variability is 0.775.

**Fig. 1:**
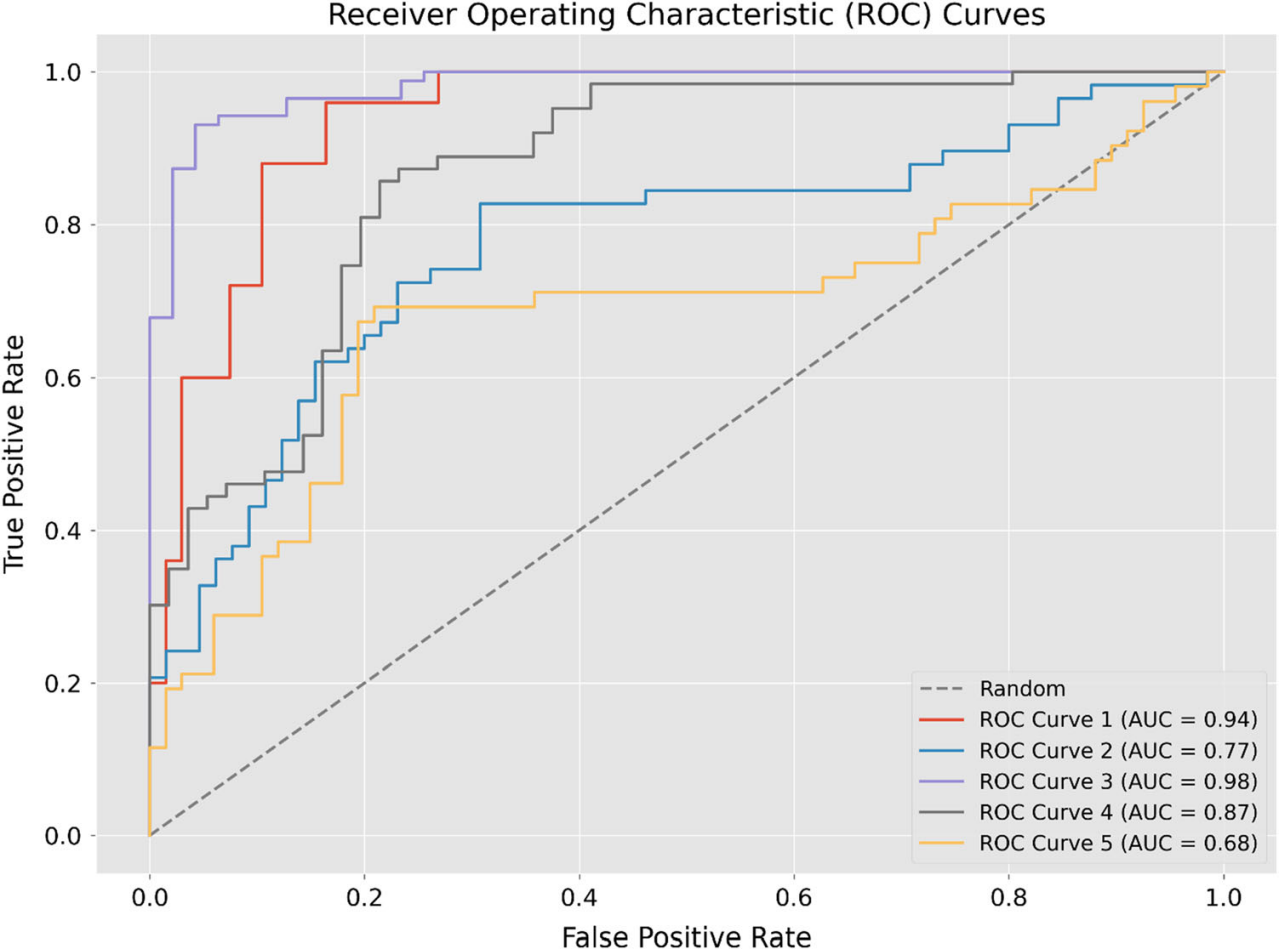
Model classification detection receiver operating characteristic (ROC) curve with five-fold analysis for confidence interval calculation

**Fig. 2:**
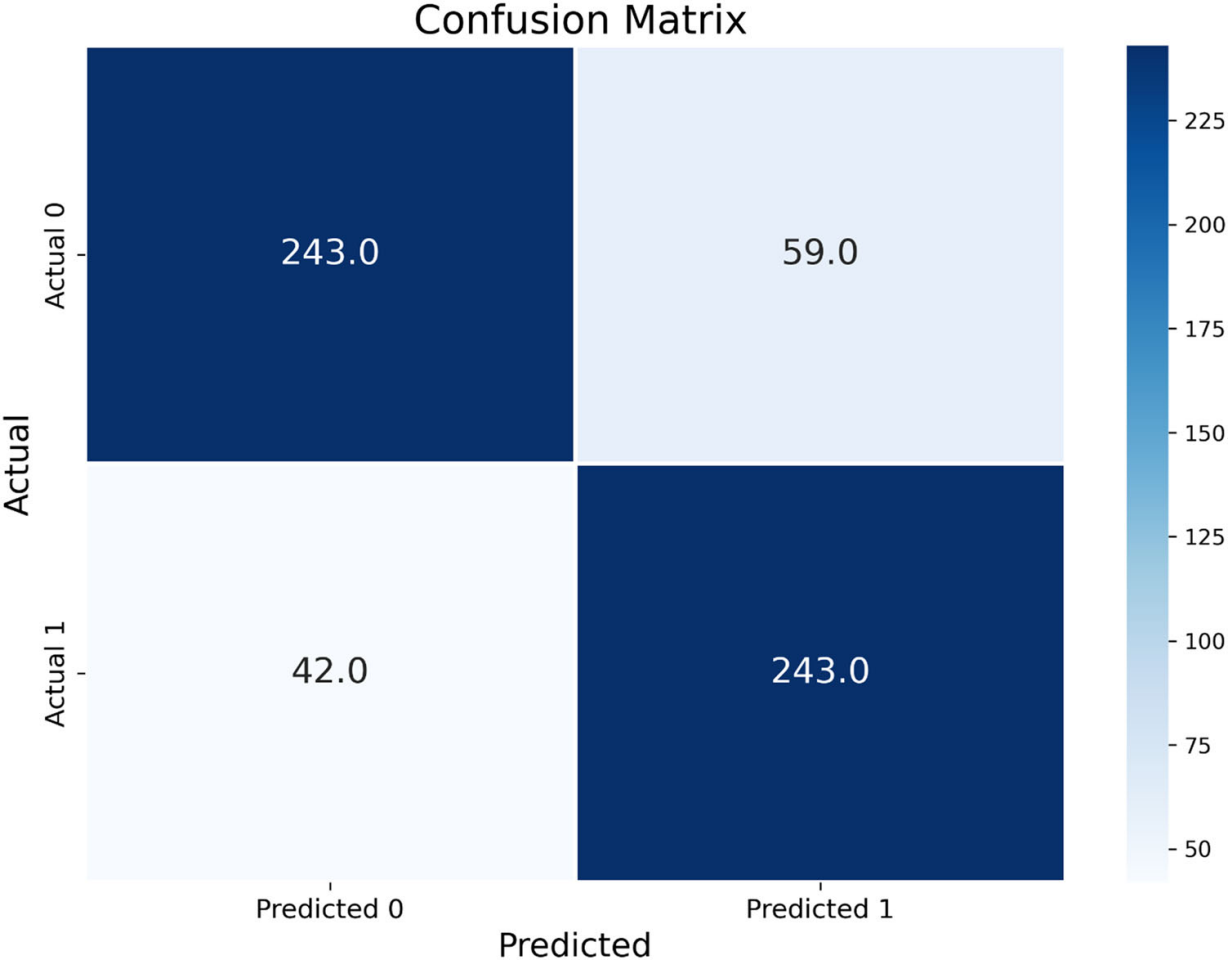
Confusion matrix comparing all the validation cohort folds using Youden’s index as cut-off value and illustrating classification results between the two image groups

After the model training and validation, the average time taken to classify a single image was 166 ms. The model’s AUC was 98.8 ± 0.3% for the training cohort, and 85.0 ± 10.9% for the validation cohort (Fig. 1). The average classification accuracy was 87.6 ± 8.3% for the training cohort and 77.3 ± 4.9% for the validation cohort. A confusion matrix comparing all the validation cohort folds using Youden’s index as cut off value is illustrated in Fig. 2.

Using Youden’s index, the model detected arterial extravasation with a sensitivity of 85.4 ± 9.4% and a specificity of 81.2 ± 8.6%. The negative predictive value (NPV) is 85.6 ± 7.3%, the positive predictive value (PPV) is 78.1 ± 10.8%, and the F1 score is 81.2 ± 8.3%.

Further analysis was performed to present the performance across various predetermined sensitivity cutoff values. When aiming for a maximum sensitivity of 99%, the classification specificity is recorded at 45.1 ± 3.4%, accompanied by an NPV of 85.4 ± 2.4%, PPV of 62.3 ± 3.4%, and F1 score of 75.3 ± 4.9%. Table 1 provides a concise summary of these findings for the validation cohorts. (Similar summary table for the training cohort can be found as supplemental data.)

**Table 1:** Performance metrics for detection of arterial bleeding in the DSA images for Youden’s index and for different sensitivity values—validation cohort.

Metric	Youden’s index	Sensitivity=90%	Sensitivity=95%	Sensitivity=99%
Sensitivity	85.4 ± 9.4%	90%	95%	99%
Specificity	81.2 ± 8.6%	59.4 ± 3.2%	52.5 ± 2.7%	45.1 ± 3.4%
PPV	78.1 ± 10.8%	69.4 ± 9.9%	66.4 ± 2.9%	62.3 ± 3.4%
NPV	85.6 ± 7.3%	76.3 ± 2.0%	82.7 ± 8.1%	85.4 ± 2.4%
Accuracy	77.3 ± 4.9%	71.8 ± 6.7%	71.3 ± 8.6%	69.0 ± 6.2%
F1	81.2 ± 8.3%	76.8 ± 6.1%	76.7 ± 8.9%	75.3 ± 4.9%

**Fig. 3:**
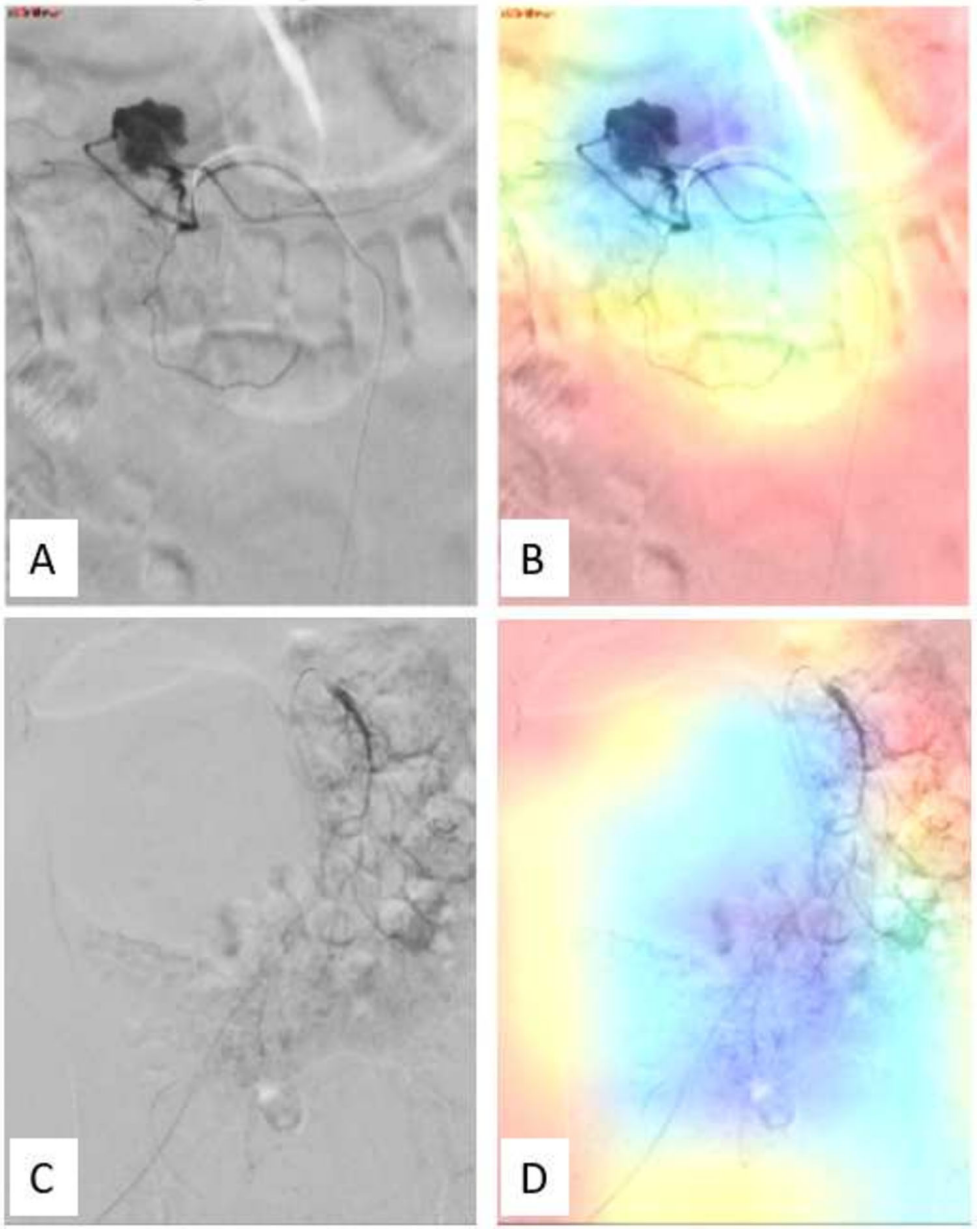
Class activation maps (CAMs) [heatmaps] of active bleeding DSA images. **A**: DSA image displaying extravasation from a branch of the celiac trunk. **B**: Fusion of the IOUS image and the final network gradients producing the heatmaps class activation map (CAM) for image A. **C**: DSA image with a subtle extravasation that was not detected by the model. **D**: Heatmap class activation map (CAM) for image C, illustrating the model’s missed detection in a challenging scenario

Class activation maps (CAMs, heatmaps) were utilized to visualize the regions of interest that influenced the deep learning algorithm’s decision-making process in categorizing images as normal or indicative of extravasation. Figure 3 presents examples of this visualization. Figure 3A shows a DSA image with clear arterial extravasation, while Fig. 3B illustrates the corresponding heatmap, where the ‘hot’ areas highlight the pixels most influential to the algorithm's decision. These areas within the site of extravasation demonstrate the algorithm’s ability to accurately identify significant regions for decision-making. Furthermore, Fig. 3C displays a DSA image with subtle extravasation that the model failed to detect, and Fig. 3D shows the heatmap for this image, indicating areas the algorithm focused on but missed the subtle signs of extravasation. This contrast between Figs. 3B and D provides insight into the algorithm’s performance across a spectrum of scenarios, from clear to subtle, underscoring its capabilities and limitations in identifying active bleeding.

## Discussion

The detection and effective management of acute nonvariceal gastrointestinal bleeding (GIB) pose significant challenges due to its diverse etiology and potential severity. Interventional radiologists are pivotal in managing patients with UGIB when conservative medical treatment or endoscopy fall short [19–23]. In this study, we aimed to enhance the detection rate of extravasation during angiographic procedures for acute UGIB using an AI model.

Certain conditions like a hypervascular bowel mucosa and bowel peristalsis or respiratory motion can lead to artifacts causing misregistration on digital subtraction angiography (DSA). Additionally, there are other angiographic findings, beyond contrast extravasation, that can provide insights into the underlying cause and source of gastrointestinal (GI) bleeding in specific pathological conditions. For instance, in cases of peptic ulcer disease, contrast collections may be observed within an ulcer crater or outlining the gastric or duodenal mucosa. In some instances, extravasated contrast may pool within the gastric rugae, bowel folds, or haustra, resembling a vein, known as the “pseudo-vein sign” [24, 25].

The AI model utilized in this study was based on the EfficientNet-B5 neural network model, which was pre-trained on the ImageNet dataset and fine-tuned using digital subtraction angiography (DSA) images. The model achieved promising results with an average validation AUC of 85.0% and an average validation classification accuracy of 77.3%.

Our findings demonstrate that the AI model, demonstrating high sensitivity, can reduce false-negative rates and assist interventional radiologists in identifying images with active bleeding during angiographic procedures. This can potentially enhance the success of transcatheter arterial embolization for managing UGIB.

The use of class activation maps (CAMs) provided valuable insights into the AI model’s decision-making process [16]. The “hot” areas within the CAMs highlighted the regions of interest that influenced the AI algorithm's classification, providing transparency and interpretability to the model’s predictions. Notably, the contrasting examples shown in our results—where the AI successfully identified clear cases of extravasation and instances where it failed to detect subtle signs—underscore the tool’s utility and limitations. This visualization tool can aid interventional radiologists in understanding the basis for the AI model’s outputs and building trust in its performance.

The implementation of AI in Interventional Radiology for GIB has the potential to streamline and optimize the clinical workflow. Previously published studies have evaluated AI models for classification of bleeding in CTA images [2–4]. By automating the detection of active bleeding sites at DSA images, interventional radiologists can focus on performing the necessary embolization procedures promptly, thereby reducing procedural time and improving patient outcomes. Additionally, the AI model’s ability to quickly analyze images at an average time of 166 ms can expedite the decision-making process and contribute to more efficient patient care.

This study has several limitations. First, the study’s retrospective nature and reliance on a single medical center's data may introduce selection bias. Second, the AI model’s performance could be further improved with additional fine-tuning on a more extensive dataset, including cases with subtle or atypical angiographic findings. Another important issue is the exclusion of images with significant motion artifacts. While this decision was made to ensure the clarity and quality of the data used for our AI algorithm, we recognize that it may impact the generalizability of our findings to all clinical scenarios. In real-world settings, DSA images with motion artifacts are not uncommon, and their exclusion could limit the applicability of our AI model in routine clinical practice. Therefore, we emphasize the importance of future studies to test and potentially adapt our AI model for use with a broader spectrum of image qualities, including those compromised by motion artifacts. This will be crucial for developing an AI tool that is robust and effective in a wide range of clinical situations. Finally, the model's external validity was not assessed, as it was trained and evaluated exclusively on images from one medical center and in a single clinical setting, potentially limiting its real-world applicability.

In conclusion, the development of an AI model for detecting arterial extravasation during angiographic procedures represents a significant step toward enhancing the management of acute nonvariceal UGIB. Our study demonstrates the potential of AI as a supportive tool for interventional radiologists, aiding in accurate and timely diagnosis, and subsequent successful embolization. As the field of AI in Interventional Radiology continues to evolve, it holds the promise of improving patient care and outcomes in a wide range of clinical scenarios. Prospective studies and real-world clinical implementation are warranted to validate and refine the AI model’s performance before its widespread adoption in clinical practice.

### Supplementary Information

Below is the link to the electronic supplementary material.

Supplementary file1 (DOCX 12 kb)
