# Supplementary material for: Artificial Intelligence for Identification of Images with Active Bleeding in Mesenteric and Celiac Arteries Angiography
Source: Cardiovasc Intervent Radiol. 2024 Mar 26;47(6):785–92. doi: 10.1007/s00270-024-03689-x (PMC11164777; doi:10.1007/s00270-024-03689-x)
Supplement: Supplementary file 1 — Supplementary file1 (DOCX 12 kb) [file 270_2024_3689_MOESM1_ESM.docx]

**Tables 1:** Performance metrics for detection of arterial bleeding in the DSA images for Youden’s index and for different sensitivity values - training cohort.

| **Metric** | **Youden's index** | **Sensitivity=90%** | **Sensitivity=95%** | **Sensitivity=99%** |
| --- | --- | --- | --- | --- |
| Sensitivity | 96.9 ± 1.1% | 90% | 95% | 99% |
| Specificity | 93.1 ± 1.2% | 96.6 ± 0.5% | 93.9 ± 1.6% | 84.8 ± 6.3% |
| PPV | 92.9 ± 1.7% | 96.1 ± 4.7% | 93.5 ± 7.3% | 86.2 ± 4.0% |
| NPV | 96.9 ± 1.2% | 90.9 ± 2.3% | 94.7 ± 0.5% | 98.3 ± 6.2% |
| Accuracy | 94.9 ± 0.8% | 93.3 ± 9.8% | 94.1 ± 2.5% | 91.5 ± 6.3% |
| F1 | 94.8 ± 1.0% | 92.9 ± 0.5% | 94.2 ± 9.6% | 92.1 ± 6.1% |
